# Child marriage in contexts of forced displacement: exploring drivers and decision-making in Jordan through a gender and generational lens

**DOI:** 10.3389/fsoc.2025.1599991

**Published:** 2025-11-21

**Authors:** Elizabeth Presler-Marshall, Nicola Jones, Sarah Baird, Bassam Abu Hamad, Sarah Alheiwidi, Erin Oakley

**Affiliations:** 1 ODI Global, London, United Kingdom; 2 Department of Global Health, George Washington University, Washington, DC, United States

**Keywords:** child marriage, gender norms, decision-making, refugee, conflict-affected, Jordan

## Abstract

**Introduction:**

Humanitarian actors have been slow to address child marriage, despite evidence that it is particularly common in conflict-affected contexts. This article explores the child marriage decision-making process among refuges living in Jordan, using a gender and generational lens.

**Methods:**

Data used in this paper was collected between 2018 and 2022 and focuses on refugee girls (and young women) who live in Jordan and who married prior to age 18. Survey data was collected from 152 young Syrian brides. In-depth interviews were conducted with 45 Syrian and Palestinian brides—as well as their parents, in-laws, and husbands.

**Results:**

Our research finds that girls' parents, grooms' parents, grooms, and girls themselves operate under deeply constrained conditions resulting from the legal and economic precarity experienced by refugee communities, and that these disadvantages reinforce gender norms and commitments to clan and culture. Girls' fathers are often beholden to their brothers to provide brides for their nephews; mothers prefer child marriage because of expectations that they will vouchsafe their children's behavior and the family's honor; grooms view marriage to girls as way of achieving adult masculinity and girls who married as children report that they felt like active agents in the process-albeit because they have so few other options.

**Implications:**

Given the importance of economic precarity, ending child marriage in contexts of forced displacement will require expanding girls' access to education and improving refugees' access to work, which will provide a route through which fathers and young men can demonstrate their adult masculinity, and to allow girls and women some measure of financial independence. Given that displacement now often lasts for decades, the humanitarian sector also needs to focus on addressing the gender norms that leave girls at risk of child marriage.

## Key messages

- Among Jordan's refugee populations, decisions about child marriage are shaped by economic and legal precarity, which reinforces gender norms and leaves all actors—fathers, mothers, girls, and grooms—feeling that their options are deeply constrained.
- Adult women are more central to marriage decision-making than has been assumed, because they are responsible for making sure their children adhere to entrenched gender norms; adolescent girls—given their very limited options—often feel they are active agents in the child marriage decision-making process; fathers' decision-making is often constrained by familial obligations.
- Interventions and messaging to counter child marriage need to be tailored, based on an understanding of this complex gender and generational “order,” and should take account of actors' social roles and developmental needs.

## Introduction

Child marriage, which each year truncates the trajectories of 12 million girls globally, is recognized as an abrogation of girls' rights and a form of gender-based violence [United Nations General Assembly, 2015; UNICEF, 2023]. However, although international and national actors have embraced efforts to end child marriage in non-conflict-related contexts, the humanitarian sector—which is focused more on meeting immediate survival needs—has been slower to do so (Murphy et al., 2016; Wodon et al., 2017; Mazurana et al., 2019; Girls Not Brides, 2022). This lack of attention is despite research which finds that rates of child marriage in conflict-affected contexts are twice the global average, and despite the ever-growing number of young people who experience protracted displacement [Human Rights Council, 2019; Singh et al., 2022; United Nations High Commissioner for Refugees (UNHCR), 2022].

This article draws on primarily qualitative data collected with Palestinian and Syrian refugees living in Jordan between 2018 and 2022. It explores how conflict and displacement leave girls (and some boys) at elevated risk of child marriage by amplifying the structural and cultural factors that support this harmful practice and introducing new forms of legal and economic precarity. It also highlights that these dynamics are best understood by examining child marriage decision-making through the eyes of the girls, grooms, and parents involved. In the next sections, we briefly review the literature on child marriage decisions, introduce the Jordan context, and describe our research methods. We then present our findings, which are organized by the actors involved in decisions about child marriage. We conclude by discussing the implications of our findings for policy and programming.

## Literature review

### Social norms and child marriage

The social norms literature provides a useful lens for understanding why child marriage continues, even as individual preferences evidence change (e.g., Cislaghi and Heise, 2019; Taylor et al., 2019; Shakya et al., 2020; Abdurahman et al., 2022; Sripad et al., 2024). Key scholars argue that social norms reflect community expectations Bicchieri et al., (2014); Mackie et al., (2015); Mackie, (2018); Cislaghi and Heise, (2019); Legros and Cislaghi, (2019). While definitions remain diverse, Bicchieri (2014)) defines a social norm as a rule of behavior that people prefer to conform to because they believe that others in the community conform to it. Moreover, they believe that others in the community expect them to conform to it, and that if they do not conform to it, then they may be socially sanctioned. Using this definition, child marriage is itself a social norm, because most people (in certain contexts) believe that most girls marry prior to age 18, they believe that others expect them (or their daughters) to marry at that time, and they believe that there will be costs if they (or their daughters) do not marry at that time Bicchieri, (2014).

### Child marriage drivers

Malhotra and Elnakib (2021)), in their systematic review of the evidence base, observe that as the economic costs of child marriage (estimated by Wodon et al., 2017 to amount to trillions of dollars) have become increasingly visible, research on the antecedents of child marriage has seen explosive growth. Quantitative research has found strong links between child marriage and other factors, including: education level of girls, boys, and parents; household size and economic status; and rural residence (Malhotra and Elnakib, 2021; Siddiqi and Greene, 2022; Edmeades et al., 2022; Pourtaheri et al., 2023). Qualitative research has focused on the social drivers of child marriage and has consistently found that it is fundamentally driven by gender inequality—even when it is boys who marry as children (Malhotra and Elnakib, 2021; see also Psaki et al., (2021); Rialet et al., (2022); Siddiqi and Greene, (2022); Pourtaheri et al., (2023). Indeed, Malhotra and Elnakib (2021)) note that gender inequality is so central to understanding child marriage that it is worth “unpacking” the ways in which it is embedded. They identify three primary pathways: beliefs about gender and sexuality (especially in relation to girls); the relationship between gender and economics; and beliefs about gender roles and how they shape broader opportunities for girls and women (Malhotra and Elnakib, 2021; see also Psaki et al., 2021; Rialet et al., 2022; Pourtaheri et al., 2023). A small but growing body of research has found that conflict and displacement amplify each of these pathways by leaving families more fearful of sexual violence and more protective of girls' honor (Bartels et al., 2018, 2020; Leigh et al., 2020; Al Akash and Chalmiers, 2021; Gausman et al., 2022; Hunersen et al., 2024; Girls Not Brides, 2022; Mieth et al., 2025). Conflict and displacement also make families more financially insecure and thus likely to use child marriage as an economic coping strategy, thereby reducing girls' opportunities for education and employment (Bartels et al., 2018, 2020; Leigh et al., 2020; Al Akash and Chalmiers, 2021; Gausman et al., 2022; Hunersen et al., 2024; Girls Not Brides, 2022; Mieth et al., 2025).

### Child marriage decision-making

Research on child marriage decision-making is far more limited, but tends to emphasize the role of parents, with fathers found to be the final decision-makers in many contexts (McDougal et al., 2018; Kakal et al., 2023; Wahyuningsih et al., 2025). Several studies have found that girls—or boys—rarely object. In part, this is because consent cannot be understood as diametrically opposed to coercion, given the strength of norms surrounding child marriage and the limited other options open to girls in contexts where child marriage remains common (Mukherjee and Sekher, 2017; Stark, 2018; Mowri et al., 2020; Lokot et al., 2021; Baraka et al., 2022; Howe et al., 2022; Lokot et al., 2022; Mieth et al., 2025; Wahyuningsih et al., 2025). In part, however, this is because parents deliberately “manufacture” consent, by encouraging adolescents to see child marriage as not just being in their own best interest but that of their family too (Dean et al., 2019). The two recent studies that have explored marriage decision-making among Syrian refugees in Jordan have both concluded that women and girls have more input into marriage timing than is often presumed, given prevailing and deeply patriarchal gender norms. They note that this is because, as discussed above, in the context of displacement, marriage can confer security and status (Zuntz et al., 2021; Gausman et al., 2022; Lokot, 2023).

### Familial violence

Conflict is not the only form of violence which shapes child marriage decision-making. Decisions must also be understood in the context of familial violence. Research has found that such violence is not siloed and that emotional and physical violence against children often occurs alongside intimate partner violence, especially in conflict-affected contexts (Hamby et al., 2010; Pearson et al., 2023; Asghar et al., 2025). It has also been found that violence is transmitted across generations, with children who grow up exposed to violence more likely to become perpetrators of myriad forms of violence themselves (Crombach and Bambonyé, 2015; Guedes et al., 2016; Fulu et al., 2017; Velloza et al., 2022).

## The Jordan context

Jordan is one of the world's least gender equitable middle-income countries. In 2024, it was ranked 123rd out of 146 countries on the Global Gender Gap Index (World Economic Forum, 2024). Although women in Jordan are better educated than men, and healthy life expectancies are not shaped by gender, women in Jordan have extremely limited access to paid work, are almost entirely excluded from politics, and have limited legal rights (World Economic Forum, 2024). For example, Jordan's Personal Status Law grants fathers and husbands control over girls' and women's movement and gives men and not women, the right to pass on citizenship to children, to file for divorce, and to have legal custody of children after divorce (Government of Jordan, 2019).

Approximately one-third of Jordan's estimated 11.3 million residents are refugees—primarily Palestinian and Syrian (Department of Statistics, 2023). Although most Palestinians in Jordan were granted citizenship long ago, nearly 20% are stateless. These families, most of whom are ex-Gazans (and their descendants) who were displaced to Jordan in the late 1960s, live in formal refugee camps and face extremely high unemployment and poverty levels (the poverty rate in Jerash camp is 53%) because they are excluded from most forms of professional work (and are required to purchase expensive work permits to undertake others) and are prohibited from accessing formal financial services and owning most types of property (Abbas and Toor, 2021).

Jordan's Syrian population (most of whom fled the Syrian civil war that erupted in 2011) primarily (~80%) live in Jordanian host communities, although a large minority live in formal refugee camps and a much smaller number live in informal tented settlements. Despite assistance from the international community, the poverty rate among Syrians is more than five times as high as that for Jordanians (80% vs. 15.7%), because the government allows them to work only in the agricultural, construction and sanitation sectors, charges them to access health and post-secondary education services, and prohibits their ownership of property and access to financial services (Ministry of Planning and International Cooperation, 2020).

Although the legal age of marriage in Jordan is 18 (for women and men), girls are allowed to marry at age 16, with the permission of a Sharia court and with the caveat that marriages must be voluntary (Human Rights Council, 2019). The most recent Jordan Population and Family Health Survey (JPFHS) found that 20% of Syrian women aged 20–24 had been married before the age of 18, and 4% had been married by age 15 [Department of Statistics (DoS) ICF, 2024]. A UNICEF study found that in 2013, nearly 18% of marriages among Palestinian refugees involved girls under the age of 18 [United Nations Children's Fund (UNICEF), 2014].

## Methodology

This article draws on data collected as part of the Gender and Adolescence: Global Evidence (GAGE) longitudinal research study, which has been using mixed-methods research to follow just over 4,000 Syrian, Palestinian and Jordanian adolescents living in five governorates of Jordan since 2018 (Baird et al., 2021). Adolescents were aged 10–17 at baseline. While most of the sample was randomly selected, adolescent girls who had been married as children were deliberately oversampled, in keeping with GAGE's focus on the most disadvantaged (Baird et al., 2021).

Data used in this paper was collected between 2018 and 2022 and focuses on refugee girls (and young women) who married prior to age 18. Survey data was collected from 152 young Syrian brides (see Table 1). In-depth interviews were conducted with 45 Syrian and Palestinian brides—as well as their parents, in-laws, and husbands (all of whom were adults when interviewed). All Palestinians in the sample are stateless and live in Jerash camp, while Syrian refugees live in host communities, formal camps, and informal tented settlements.

**Table 1:** Research sample.

**Quantitative sample**	**Qualitative sample**
•152 married Syrian girls •Residence location: host communities (64%), formal UNHCR camps (30%), informal tented settlements (7%) •Mean age when interviewed: 16.7 years •Mean highest grade attended: 7th •Mean age when married: 15.4 years •Mean age gap with husband: 5.8 years	•45 girls (and young women) who married prior to age 18 •38 Syrians (25 in host communities, 10 in formal camps, 3 in informal tented settlements); 9 Palestinians •Age when interviewed: 14–22 •Age when married: 11–17 •Age gap with husband: 2–15 years •Educational attainment: grade 2 to grade 10

GAGE research tools consisted of a broad survey, which included a module on marriage, and an array of interactive activities, including vignette-based discussions, social norm mappings, timelines, and a child marriage decision-making tool (the tools are available online) (Baird et al., 2019; Jones et al., 2018, 2019). The tools were employed by carefully trained researchers of the same sex, and the same nationality, as the respondent. Statistical analysis was conducted using Stata 18.0. Qualitative data analysis involved a multi-step process. Preliminary analysis took place during daily debriefings with the research team to discuss similarities and differences across sites; these findings were used to develop a thematic codebook informed by GAGE's conceptual framework, based on a gendered capabilities approach that allows us to explore young brides' broader lives and the socio-ecological forces that shape them (GAGE Consortium, 2019). All interviews were transcribed and translated by native speakers of the local language, then coded using a qualitative software package, MAXQDA.

The research design and tools were approved by the George Washington University Committee on Human Research's Institutional Review Board (071721), ODI Global's Research Ethics Committee (02438), and all appropriate ethics review boards at national (and camp) levels. Consent (written or verbal as appropriate) was obtained from caregivers and married adolescents; written or verbal assent was obtained for all unmarried adolescents under the age of 18. Care was taken, given the sensitive nature of the questions, to communicate the objectives of the research to young brides as well as their natal and marital family members. There was also a robust protocol for referral to services, tailored to individual research sites based on GAGE's longstanding relationship with local community-based organizations.

## Findings

We first present survey findings on how girls' marriages were transacted and then turn to qualitative findings on why actors choose child marriage. Respondents' preferences for child marriage are best understood—and become more actionable for policy and programming—when respondents are categorized based on their social roles. With the caveat that we triangulate preferences using insights from both actors themselves, as well as from other family members (because social norms often keep actors from recognizing and articulating their own thought processes), results are organized accordingly.

The GAGE baseline survey found that child marriages are often hasty, but rarely forced and usually not unwanted by the girls entering into them. Just over one-quarter (27%) of young brides reported that they had known their husband for less than a month before their wedding; 11% had known him for less than a week (see Table 2). Despite this haste, 94% of girls reported having married voluntarily and 63% reported having been ready to marry when they did.

**Table 2:** Characteristics of young brides' marriages.

**Length of time young brides had known their husband prior to marriage**	**•Less than a year = 76%** **•Less than a month = 27%** **•Less than a week = 11%**
Type of marriage	•Voluntary = 94% •Forced = 6%
Young brides' readiness to marry at the time	•Ready to marry at the time = 63% •Would have preferred to wait = 37%

### Brides' fathers

Although respondents agreed that fathers are usually the final arbiters of when and to whom their daughter marries, our findings suggest that this generalization obscures significant complexity. A minority of child marriages were unilaterally decided by fathers. In almost all cases where this was true, these matches were consanguineous, and, in many cases, they went ahead despite strenuous objections by the girl and her mother. Although fathers themselves insisted that they prefer that their daughters marry paternal cousins to keep the girls safe, young brides and their mothers were unanimous that fathers' decisions are less about fathers' fears for girls' safety and more about fathers' fears for their own role in the extended paternal family. A 19-year-old Syrian young mother recalled that her future had been the coin with which her father had bought family harmony:

“*My uncle told him that there would be hatred if we refused the marriage*.”

Respondents noted that conflict and displacement have strengthened fathers' commitment to consanguineous marriage, because displacement has limited the economic opportunities open to the young men who are their nephews (and who are allowed to proffer a lower bride price because they are related), and has exacerbated parental fears about their sons' behavior. A Syrian father reported that while he would have preferred to wait until his daughter was 18 before she married his brother's son, his brother refused to wait because he was afraid that his son would shame the family by engaging in a premarital relationship:

“*My brother… he is in a hurry because his son is 20 years old*.”

Most fathers reported that marriage decisions were made after consultation with their wives, despite admitting that this had limited value to them, because they see themselves as “rational” heads of household. A Syrian father explained,

“*You take her advice, of course, if her advice is sound. But a woman always gives her opinions with her affection*.”

In some cases, fathers explained that their support for child marriage was because of their role as the family breadwinner, as they feel most acutely the pressure to ensure household food security in the context of high unemployment and very limited work opportunities for refugees. Palestinian respondents—who have endured generations of exclusion from the labor market and have effectively no access to formal social protection—were the most likely to report that girls are pushed into child marriage to improve the living standards of their natal family. A Palestinian mother explained,

“*When the father can't cover expenses, he will find himself forced to marry his daughters to reduce expenses*.”

In other cases, fathers' support for child marriage was rooted in their responsibility for protecting the honor of the family and clan, which has taken on added importance during displacement because of men's restricted work opportunities and ability to provide for their families. A Syrian father reported that he married his daughter when she turned 16 to prevent her from adopting “modern” Jordanian beliefs and behaviors:

“*The girl has… departed from our customs and traditions… so that you say that you do not want your daughter to be exposed to this*.”

Fathers also reported that conflict and displacement have shifted the way they evaluate marriage as a mechanism to ensure their daughters' longer-term social and economic security. A Syrian father explained that he will marry his daughter as soon as she turns 16, so that if he is deported, his daughter will be provided for since her husband will become her legal guardian:

“*This country is not ours… We always live in fear… I may bother the authorities. Thus, they can expel me to the border*.”

Some fathers admitted that they had been pushed by others (including their own wives, the girl's future in-laws, and the broader community) into allowing their daughters to marry as children, despite having reservations about it. The father of a married Syrian girl explained that his daughter's now grandmother-in-law had worked for months to convince him to allow his daughter to marry. He recalled,

“*I told her*, ‘*The girl is too young. She still cries over a quarter of a dinar!*' *But she replied*, ‘*Do not break me… I will neither drink tea nor drink coffee until you do as I request*.”'

The mother of a married Syrian girl explained that fathers who delay their daughter's marriage are taunted by the community for failing to recognize that girls' value lies in early marriage and motherhood:

“*We say that he wants to preserve his daughter like pickles*.”

Respondents noted that pressure on Syrian fathers has increased as displacement has become protracted, and as other actors in girls' lives develop stronger preferences for child marriage (see below).

Largely absent from the existent evidence base, our research is finding that for some Syrian fathers, their role as head of household also affords them the opportunity to protect their daughters from child marriage. This is most common in formal refugee camps, where exposure to NGO programming has been regular and sustained and where the links between child marriage and divorce are highly visible (because households move less often). In some cases, fathers simply refuse to marry their daughters until they turn 18; in other cases, they delay marriage, waiting until their daughters are more mature. For example, the father of a Syrian girl who married at age 17 reported that he afforded her an extra 3 years of childhood:

“*From the 8th grade, the grooms began to propose to marry her… More than 20 young men proposed to marry my daughter… Whoever asked me to marry her, I refused because she was young*.”

### Brides' mothers

Mothers' input into child marriage decision-making is generally framed as shared with fathers, but we again find considerable complexity. In line with previous research on marriage decision-making in Jordan (e.g., Zuntz et al., 2021; Lokot, 2023), most mothers and married girls disagreed with fathers' accounts and instead reported that mothers had played the primary role in child marriage decision-making. A Syrian mother, whose daughter was married at age 14, recalled,

“*It was me* [who decided]. *Her father was refusing the marriage concept, and he used to refuse whoever proposed*.”

Fathers often added that this generally reflects mothers' role in choosing who (and consequently when) girls should marry. A Syrian father explained,

“*We are men; most of our time is outside the house. Who is the closest person to the girl?*”

It was not uncommon for mothers to report having put considerable pressure on their husbands to allow their daughter to marry. In some cases, this was because mothers hoped that marriage would materially improve their daughter's life. This was most common among Syrians, the majority of whom recall higher living standards pre-conflict. A Syrian mother explained,

“*The girl needs many requirements, and the mother doesn't have the capability to provide for the girl's needs… I can let my girl stay with me until the age of 18 or 19, but I can't see my girl not dressing like other people… So, when a good man wants to marry her, I consider this as a chance. I say:* ‘*Let me marry her to this man to bring her what she wants*.”'

In other cases, mothers' pressure on fathers reflects their concern that if girls do not marry early—before there is any possibility that their honor might be questioned—then they might be shut out of the marriage market altogether, and left as a spinster without support or status, given the limited opportunities afforded to women—especially refugee women—in Jordan. A Palestinian mother, after first noting that sexual harassment has become rampant in recent years, stated,

“*Families want to marry their daughters quickly because they are worried about them*.”

A 16-year-old Palestinian girl who had married at age 15 added,

“*My mother feared that I would never get married and get old*.”

Mothers, like fathers, also choose child marriage to ensure that girls do not stray from tradition, which has become more likely in Jordan's increasingly mixed population. A 17-year-old Syrian girl from an informal tented settlement reported that she had fallen in love with a Palestinian man with whom she worked, but her parents quickly forced her to marry a cousin she had never met instead:

“*He* [the Palestinian man] *wanted to get engaged to me, but my mother refused. My mother said that they refused to marry their girls to Palestinian men. We marry our cousins*.”

Most mothers acknowledged that they are under considerable pressure from other women in the community to ensure their daughter's honor and status—and thus their own status as a good mother—by conforming to social expectations regarding child marriage. The mother of a Syrian girl who married at age 16 explained that this pressure can leave even mothers who are opposed to child marriage believing that it is the right thing to do:

“*They say*, ‘*Do you want her to sit with you? Do you want to bear her sin?*' *So, you will be convinced by their talk and you will say to yourself:* ‘*It would be wrong to NOT marry her. It is very important that I marry her now!*”'

Mothers also sometimes work to protect their daughters from child marriage. Several young women studying at university (and drawn from the broader GAGE sample of unmarried young people) reported that their mother had been their staunchest ally, refusing to allow them to marry, and working for years to allay their father's fears about their lack of marriage. A 20-year-old Syrian young woman explained, “*My mom used to refuse, she used to convince him*.” Other girls, usually those married to a cousin, noted that their mother's efforts had been unsuccessful because of fathers' obligations to their brothers and the broader clan. A young Syrian mother who married at age 15 explained, “*I know a mother* [who] *refused to marry her daughter… Her husband beat her*.”

### Grooms' parents

Grooms' parents also emerged as key actors in the child marriage decision-making process. As noted earlier, fathers put pressure on their own brothers to provide a suitable and affordable bride for their son, which has become more important given that so few young men from refugee communities have access to stable and decently remunerated employment in Jordan. Mothers are largely in charge of initiating individual marriage matches for their sons. A 17-year-old Syrian girl who married at age 14 recalled that her mother had been randomly approached by her now mother-in-law:

“*My mother was in the market, his mother asked her* ‘*Do you have daughters for marriage?*”'

Respondents reported that grooms' parents have many reasons to want their sons married. Some are concerned that their son might begin an illicit love affair, damaging family honor, or propose to a girl they deem inappropriate (usually because she is of the “wrong” nationality or from the “wrong” clan). Both have become more likely as men's age of marriage has crept upward in response to high unemployment and poverty rates. Most parents, however, are hoping that marriage will make their sons mature and conform to dominant masculine norms. A Syrian father explained,

“*We have a quote that you get your son married so that he becomes a man*.”

Parents' concerns about their sons' transition from boyhood into manhood have grown during displacement (which for Palestinians is now decades long), because refugee boys and young men are largely excluded from secondary and post-secondary education (due to real and opportunity costs) and are confined to the margins of the labor market. This leaves them with no clear pathway to assume adult status, and with too much time on their hands. A Palestinian mother, when asked why so many boys and young men are using substances and fighting in the streets rather than settling down and contributing to the community, replied that everything comes down to the fact that “*the boy does not have a job*”. Syrian parents, while not yet evidencing the chronic hopelessness of their Palestinian peers, reported that they want their sons settled and off the streets to avoid them attracting the attention of Jordanian authorities, which might put them at risk of deportation and forced military service in Syria. A father explained,

“*If they are bothered by my son, they will send him to Syria, then he will be conscripted*.”

Grooms' parents, especially mothers, also want their sons to marry so that they can father children (preferably boys) to continue the paternal line. A 19-year-old Syrian young father explained that producing a daughter had not been sufficient to quiet his mother:

“*My mother always wanted me to get married and have children… When I had a girl child, she was upset because she wants boys and is pressuring me to remarry another girl*.”

This desire for grandchildren appears particularly common where family members have died during conflict, which creates not only an emotional gap in the mother's life but also a pressing interest in repairing the family tree. The mother of a Syrian boy, who married at age 16 to a 13-year-old girl, explained:

“*The man of the house is dead, may he rest in peace. I wanted to celebrate something… So I suggested to let him* [my son] *get married*.”

Key to understanding the role of grooms' parents in perpetuating child marriage is that they (and again, mothers especially) prefer for their sons to marry girls and not women. Respondents agreed that this preference is in part because younger brides are more likely to be “pure”, with their reputations unsullied by gossip. They also agreed that it is primarily, however, about the mother-in-law's desire for control. The Syrian mother of a girl who married at age 16 explained how,

“*The mother-in-law wants to impose her opinion and her thoughts*.”

Married girls added that these opinions and thoughts often center around how girls should cater to the needs of their new husband, who has often exhausted his own mother with years of incessant demands. A pregnant 15-year-old Syrian girl stated:

“*She* [her mother-in-law] *married her son to find a woman to bathe him. I have to cook food for him, and I have to make coffee and tea for him. I have to do everything he wants*.”

### Grooms

Boys and young men, as the other half of young couples, also play an important role in child marriage decision-making, albeit generally with the help of their mother. In a small but increasing number of cases, grooms are themselves initiating marriage matches with girls. Some grooms (almost always those entering their mid-20s) approach non-related girls on their own. For example, a 17-year-old Syrian girl noted that she was only 12 when her now husband (then aged 23) had picked her out, bought her a phone, and “*made me feel that he cared about me*”. While her father “*refused every time*” the man wanted to visit, her mother helped her hide the phone, so that she could text the man. She was married at age 13. In most cases, generational hierarchies mean that even young men marry as their parents direct them to. A 19-year-old Syrian young woman who was married at age 17 to a man aged 25 at the time reported that her husband—refused permission by his own parents to marry the girl he loved—had given up:

“*He told his parents:* ‘*It's up to you. You can choose whoever you want*.”'

Respondents offered several explanations for why boys and young men either initiate or capitulate to marriage arrangements. Unsurprisingly, given that males and females are allowed little contact with one another after puberty, the most common explanation was so that boys and young men could explore their own romantic and sexual feelings, which are increasingly recognized by media-savvy young people as normal but are still seen by adults to threaten family honor unless confined to marriage. A 16-year-old Syrian boy explained,

“*If you have a girlfriend, people will talk about you and utter rumors*.”

Indeed, most young husbands—all married to adolescent girls but not adolescents themselves—-reported that they had been pressured by parents and religious leaders to marry, to make sure they did not commit “*haram*” (forbidden) acts such as engaging in premarital sexual activity. One stated: ‘

‘*There is encouragement… not once was there was a sheikh* [Muslim religious leader] *against early marriage*.”

Other respondents noted that boys and young men choose marriage to enhance their social status. In some cases, this is so that they can impress their peers, and it reflects age-related developmental imperatives. A Syrian mother reported that her son chose to marry early so that he could capture a particularly beautiful girl:

“*He insisted on marrying this girl, the girl is beautiful and there are a lot of young men wanting to marry her*.”

In other cases, boys and young men choose to marry because it is the only pathway through which they can claim adult status, given their exclusion from higher education and better-paid sectors of the labor market. An 18-year-old Syrian father, who (somewhat unusually) married at age 16, stated that he is happy to be contributing to his family after losing many relatives during the conflict:

“*I am happy I got married…* [I am] *proud that I worked at a young age and started a family*.”

Young men were often forthcoming about their preference for a child bride, which, like their mother's preference, revolved around greater certainty about the girl's purity and their own need for control. The husband of a Syrian girl who married at age 15 reported that concerns about girls' virginity have been so heightened since displacement that some religious leaders—in direct opposition to Jordanian law (which allows religious exemptions for girls over the age of 16)—are encouraging child marriage:

“*The sheikh said to marry a girl of 15 years*.”

The 25-year-old husband of a 15-year-old Syrian girl added that choosing a younger wife also ensures the compliance that allows men to feel powerful—a rarity in an environment rife with legal and economic insecurity:

“*I raised her, knew everything about her, and made her know everything about me while she is still young… It's like the doll, you can move it as you like… It's better than getting a mature girl who already has other things*.”

### Young brides

A minority of young brides reported having been genuinely enthusiastic about their own child marriage. For these girls, explanations highlighted adolescent developmental imperatives and how those play out in communities where girls are increasingly aware of options, and yet those options remain off-limits. For example, girls reported having married for love (because they watch romantic television for hours each day), to alleviate boredom (because they are not allowed to leave home even for school), or to claim status among their similarly aged female cousins (because other forms of status are largely unobtainable). One 19-year-old Syrian young woman who had married at age 16 admitted that she had simply “*wanted to have a party and wear a dress*” and have something nice in her otherwise deprived life. Girls noted that displacement has made child marriage a more attractive option because it has heightened parents' concerns about girls' honor and tightened parents' restrictions on their daughters' lives. A 17-year-old Syrian girl, pointing to a photo of her mother when she was young, stated that:

“*During my mother's time, women enjoyed more freedoms and mobility… Today, girls are denied their freedom… This was all a result of the war and displacement, and the rumors about girls that people circulate in our community*.”

With the caveat that consent cannot be understood as diametrically opposed to coercion, given the strength of norms surrounding child marriage (Mowri et al., 2020), most young brides' broader narratives suggest that they felt ready to marry because their consent had been “manufactured” by their parents (Baraka et al., 2022). In some cases, fathers had worked to convince girls that child marriage was in their own best interests. A 16-year-old Syrian girl reported that,

“*My father said it is better to marry because he… is an old man… After that, I agreed to marry*.”

However, girls were more likely to report coaching from their mother, who was often able to convince the girl that she was in love with a man she had only just met. A 14-year-old Syrian mother, who was married at age 13 to a man she had never met but whose mother had approached her own mother, recalled having been madly in love:

“*I loved him at first sight… I wanted to get married*.”

Parents—one of whom claimed:

“*we don't force the girl to get married*”

(mother of a Syrian girl from an informal tented settlement, who married at age 15)—appear to know exactly what they are doing. A young Syrian bride's mother admitted:

“*My daughter said that she didn't want to marry since the beginning… I played with her mind*.”

Families can be so effective at convincing their daughters that they want to marry that even the judicial procedures meant to stop child marriages are unsuccessful. A 15-year-old Syrian girl, married to a 26-year-old man, recalled:

“*A woman* [from the court] *asked me why I want to marry early and told me that I'm still young for responsibility. I told her that I can handle this responsibility*.”

Many young brides, like their Rohingya peers who took part in Mieth et al.'s (2025)) research, reported that they had agreed to marry because they were capitulating to the inevitable. Sometimes this was to improve the lives of their younger siblings, by freeing up household resources; sometimes to silence the community gossip that surrounds girls, especially once they have left school. Other times it was because displacement has narrowed (or eliminated) other pathways through which girls might achieve adult status or because girls are socialized to be docile and compliant. A 17-year-old Syrian girl explained that she was married as a child because secondary and post-secondary education are unaffordable to refugees in Jordan:

“*All my older sisters continued their education… back in Syria. It was different for me. I could not continue my education… They married me off when I reached 16*.”

A now-divorced 16-year-old Syrian girl observed that she had married when her mother told her it was time to marry because

“*We don't like to say no to my mother*.”

Girls have the least space to gainsay a child marriage when they are to marry a cousin, many of whom are almost completely unknown to girls, given that both extended families and marital age gaps are large—because they are terrified to open rifts in the extended family. A 19-year-old Syrian young woman who married a cousin at age 17 recalled,

“*I wanted to say no but I couldn't… because of the traditions*.”

A few girls (all Palestinian) admitted that they had capitulated and given formal consent to a forced child marriage because they had been threatened by their parents with bodily harm. A divorced 16-year-old reported that although she had cried and pleaded with her father, who had arranged a marriage on 5 days' notice, she then lied to the judge when he asked if she was being forced to marry, to save face in the courtroom:

“*I said no* [to the judge] *because my father was with me*.”

Critically, in terms of understanding why girls agree to marry as children—whether willingly or because they are resigned to their fate—respondents noted that girls are only rarely given enough information about marriage to make “no” seem a desirable answer. Indeed, although young brides knew they would gain a husband and move into a marital home where they would be expected to take on household work, many apparently knew little about the broader responsibilities (emotional and sexual) involved in being a wife. A 16-year-old Syrian girl, married at age 13 and now trapped in an abusive marriage, recalled:

“*I did not know anything. All I was happy about was the dress and the gold back then, ha ha!*”

## Limitations and strengths

This research is limited in that it draws on the experiences of a small number of young brides who married as children. Therefore, results may not be generalizable. However, it is strengthened by being situated inside a much larger research program, which provides ample opportunity for triangulating actors' responses with those of unmarried young people, parents who have eschewed child marriage for their children, and key informants (ranging from educators to religious leaders).

## Conclusion and implications

Our findings regarding broader explanations for child marriage are in line with existent research and emphasize the importance of concerns about sexuality and honor, the role of economic precarity, limits on opportunities, and how these are exacerbated by conflict and displacement (Gausman et al., 2022; Bartels et al., 2020; Malhotra and Elnakib, 2021). They diverge from other research, however, in that they personalize these themes and explore how they play out in the decision-making of the various actors involved in perpetrating child marriage. Indeed, our findings highlight that not only do mothers (of brides and grooms) often play the central role in arranging child marriage (despite men's titular role as head of household), but that men's own decision-making is deeply constrained by loyalty to family and clan. Moreover, parents' concerns about their unmarried sons' behavior run nearly as deep as their concerns about their unmarried daughters' honor.

Among Jordan's Palestinian and Syrian refugee populations, the decision to marry children early is complex. In line with broader scholarship that argues that gender norms and “social practice” are “ordered” based on dynamic and context-specific power relations underpinned by age, class, and other power structures (Connell, 2005; Grabska, 2014), our research finds that girls' parents, grooms' parents, grooms, and girls themselves are constrained by gender norms born of deep-seated gender inequality—and fear of social sanctions—as well as legal and economic marginalization stemming from displacement. This marginalization has numerous consequences: it creates barriers to education that would strengthen young people's voices and open up new opportunities; it hinders access to decent work that would provide adult men and women (and adolescent girls and boys) with incomes and a sense of self-worth; and it strengthens adults' loyalties to the clan traditions that leave girls most at risk of child marriage.

The implications of our research for programming and policy are multi-pronged. First, given the importance of economic precarity, ending child marriage in contexts of forced displacement will require expanding young people's access to education, at least through to the end of secondary school. This should be coupled with stepped-up social protection and improved access to the labor market to relieve financial pressure on households, provide a route through which fathers and young men can demonstrate their adult masculinity, and to allow girls and women some measure of financial independence. As was trialed in Jordan (Abu Hamad et al., 2025), donors might provide cash transfers labeled for education to simultaneously improve access to education and household incomes.

Equally important, given that displacement now often lasts for decades, the humanitarian sector needs to focus on addressing the gender norms that leave girls at risk of child marriage. Interventions should be tailored to girls, parents, and prospective grooms; they should acknowledge actors' social roles and developmental needs; and they should provide information, support, and access to role models who embody alternative femininities and masculinities. Because of the cultural value placed on sexual purity—especially for girls—it is also vital to engage with faith leaders to raise their awareness of the risks of these harmful norms and to support any who champion an end to the practice of child marriage.

## Data Availability

The datasets presented in this article are not readily available because GAGE does not publicly disseminate qualitative data. Quantitative data is available upon request. Requests to access the datasets should be directed to gage@odi.org.uk.

## References

[B1] Abbas M. H. Toor S. S. (2021). Socio-economic Assessment and Practices in Jerash Camp. Amman: UNICEF Jordan.

[B2] Abdurahman D. Assefa N. Berhane Y. (2022). Parents' intention toward early marriage of their adolescent girls in eastern Ethiopia: a community-based cross-sectional study from a social norms perspective. Front. Glob. Women's Health 3:911648. doi: 10.3389/fgwh.2022.911648 36276231 PMC9581299

[B3] Abu Hamad B. Jones N. Abuhamad S. Baird S. Oakley E. (2025). Can social protection contribute to social connectedness in contexts of forced displacement and crisis? Lessons from Jordan's labelled cash transfer for education. World Dev. 188:106886. doi: 10.1016/j.worlddev.2024.106886 40171045 PMC11803516

[B4] Al Akash R. Chalmiers M. A. (2021). Early marriage among Syrian refugees in Jordan: exploring contested meanings through ethnography. Sex. Reprod. Health Matters 29:287–302. doi: 10.1080/26410397.2021.2004637 34873990 PMC8654413

[B5] Asghar K. Christofides N. Falb K. Jones N. Meinck F. (2025). Characterizing typologies of overlapping IPV and VAC in the home: findings from DRC, Ethiopia, and South Africa. Child Abuse Neglect 169:107637. doi: 10.1016/j.chiabu.2025.107637 40812143

[B6] Baird S. Camfield L. Haque A. Jones N. Anas M. Pincock K. . (2021). No one left behind: using mixed-methods research to identify and learn from socially marginalised adolescents in low- and middle-income countries. Eur. J. Dev. Res. 33:1163–1188. doi: 10.1057/s41287-021-00436-7

[B7] Baird S. Hicks J. Jones N. Kilburn K. Małachowska A. Muz J. . (2019). Jordan Baseline Survey 2017/2018: Core Respondent Module. London: Gender and Adolescence: Global Evidence.

[B8] Baraka J. Lawson D. Schaffnit S. Wamoyi J. Urassa M. (2022). Why marry early? Parental influence, agency and gendered conflict in Tanzanian marriages. Evol. Hum. Sci. 4:E49. doi: 10.1017/ehs.2022.46 37588904 PMC10426069

[B9] Bartels S. A. Michael S. Bunting A. (2020). Child marriage among Syrian refugees in Lebanon: at the gendered intersection of poverty, immigration, and safety. J. Immigr. Minor. Health 23, 472–487. doi: 10.1080/15562948.2020.1839619

[B10] Bartels S. A. Michael S. Roupetz S. Garbern S. Kilzar L. Bergquist H. . (2018). Making sense of child, early and forced marriage among Syrian refugee girls: a mixed methods study in Lebanon. BMJ Glob. Health 3:e000509. doi: 10.1136/bmjgh-2017-000509 29515914 PMC5838398

[B11] Bicchieri C. (2014). Norms in the Wild: How to Diagnose, Measure and Change Social Norms. Cambridge: Cambridge University Press.

[B12] Bicchieri C. Lindemans J. W. Jiang T. (2014). A structured approach to a diagnostic of collective practices. Front. Psychol. 5:1418. doi: 10.3389/fpsyg.2014.01418 25538666 PMC4257103

[B13] Cislaghi B. Heise L. (2019). Gender norms and social norms: differences, similarities and why they matter in prevention science. Perspect. Psychol. Sci. 15, 3–20. doi: 10.1111/1467-9566.13008 31833073 PMC7028109

[B14] Connell R. W. (2005). Masculinities. Berkeley and Los Angeles, CA: University of California Press.

[B15] Crombach A. Bambonyé M. (2015). Intergenerational violence in Burundi: experienced childhood maltreatment increases the risk of abusive child rearing and intimate partner violence. Eur. J. Psychotraumatol. 6:26995. doi: 10.3402/ejpt.v6.26995 26679146 PMC4696461

[B16] Dean L. Obasi A. El Sony A. Fadul S. El Hassan H. Thomson R. . (2019). “He is suitable for her, of course he is our relative”: a qualitative exploration of the drivers and implications of child marriage in Gezira state, Sudan. BMJ Glob. Health 4:e001264. doi: 10.1136/bmjgh-2018-001264 31263579 PMC6570976

[B17] Department of Statistics (2023). Available online at: https://dosweb.dos.gov.jo/ (Accessed March 24, 2025).

[B18] Department of Statistics (DoS) and ICF (2024). Jordan Population and Family Health Survey 2017–18. Amman and Rockville, MD: DOS and ICF.

[B19] Edmeades J. D. MacQuarrie K. Acharya K. (2022). Child grooms: understanding the drivers of child marriage for boys. J. Adol. Health 70, S54–S56. doi: 10.1016/j.jadohealth.2021.08.016 35184832

[B20] Fulu E. Miedema S. Roselli T. McCook S. Chan K. L. Haardörfer R. . (2017). Pathways between childhood trauma, intimate partner violence, and harsh parenting: findings from the UN Multi-country Study on Men and Violence in Asia and the Pacific. Lancet Glob. Health 5, e512–e522. doi: 10.1016/S2214-109X(17)30103-1 28395846

[B21] GAGE Consortium (2019). Gender and Adolescence. Why Understanding Adolescent Capabilities, Change Strategies and Contexts Matters, Second Edn. London: Gender and Adolescence: Global Evidence.

[B22] Gausman J. Huda F. A. Othman A. Al Atoom M. Shaheen A. Hamad I. . (2022). Girl child marriage and the social context of displacement: a qualitative comparative exploration of Syrian refugees in Jordan and Rohingya refugees in Bangladesh. BMC Public Health 22:2417. doi: 10.1186/s12889-022-14832-z 36550423 PMC9780094

[B23] Girls Not Brides (2022). Child Marriage in Conflict and Crisis Settings. Available online at: https://www.girlsnotbrides.org/documents/2232/Child_marriage_in_conflict__crisis_settings.pdf (Accessed September 30, 2025).

[B24] Government of Jordan (2019). Jordan: Personal Status Law No. 15 of 2019. Available online at: https://www.refworld.org/legal/legislation/natlegbod/2019/en/123423 (Accessed October 5, 2025).

[B25] Grabska K. (2014). Gender, Home and Identity: Nuer Repatriation to Southern Sudan. Rochester: James Currey. doi: 10.1515/9781782043805

[B26] Guedes A. Bott S. Garcia-Moreno C. Colombini M. (2016). Bridging the gaps: a global review of intersections of violence against women and violence against children. Glob. Health Action 9:31516. doi: 10.3402/gha.v9.31516 27329936 PMC4916258

[B27] Hamby S. Finkelhor D. Turner H. Ormrod R. (2010). The overlap of witnessing partner violence with child maltreatment and other victimizations in a nationally representative survey of youth. Child Abuse Neglect 34, 734–741. doi: 10.1016/j.chiabu.2010.03.001 20850182

[B28] Howe K. Stites E. Moran M. Marshak A. Hammada K. Sulaiman S. . (2022). Perspectives on Early Marriage: The Voices of Female Youth in Iraqi Kurdistan and South Sudan Who Married Under Age 18. Boston, MA: Tufts University.

[B29] Human Rights Council (2019). Child, Early and Forced Marriage in Humanitarian Settings: Report of the United Nations High Commissioner for Human Rights. Geneva: Human Rights Council.

[B30] Hunersen K. Jeffery A. Karim L. S. Gambir K. Metzler J. Zedan A. . (2024). Child marriage and displacement: a qualitative study of displaced and host populations in the Kurdistan Region of Iraq. J. Refug. Stud. 37, 324–335. doi: 10.1093/jrs/feae020

[B31] Jones N. Camfield L. Coast E. Samuels F. Hamad B. A. Yadete W. . (2018). GAGE Baseline Qualitative Research Tools. London: Gender and Adolescence: Global Evidence.

[B32] Jones N. Presler-Marshall E. Małachowska A. Jones E. Sajdi J. Banioweda K. . (2019). Qualitative Research Toolkit to Explore Child Marriage Dynamics and How to Fast-Track Prevention. London: Gender and Adolescence: Global Evidence.

[B33] Kakal T. Kok M. Jawad M. (2023). “You are a child and this is not your business”: decision-making on child marriage in Sindh, Pakistan. PLoS ONE 18:e0266865. doi: 10.1371/journal.pone.0266865 37773957 PMC10540960

[B34] Legros S. Cislaghi B. (2019). Mapping the social-norms literature: an overview of reviews. Glob. Public Health 14, 1479–1494. doi: 10.1080/17441692.2019.1594331 30895892

[B35] Leigh J. Baral P. Edmier A. Metzler J. Robinson C. Skanthakumar T. (2020). Child Marriage in Humanitarian Settings in South Asia: Study Results from Bangladesh and Nepal. Kathmandu: UNFPA Asia Pacific Regional Office (APRO) and UNICEF Regional Office for South Asia (ROSA).

[B36] Lokot M. (2023). Decision-making, violence, resistance, and love: contested and complicating narratives of Syrian marriages. Violence Against Women 30, 31–53. doi: 10.1177/10778012231207037 37822250 PMC10666506

[B37] Lokot M. Shakya H. Cislaghi B. (2022). The limits of child agency?: dissonances and contradictions in conceptualisations of agency within child-led marriages in Somalia and Cameroon. Int. J. Children's Rights 30, 173–203. doi: 10.1163/15718182-30010007

[B38] Lokot M. Sulaiman M. Bhatia A. Horanieh N. Cislaghi B. (2021). Conceptualizing “agency” within child marriage: implications for research and practice. Child Abuse Neglect 117:105086. doi: 10.1016/j.chiabu.2021.105086 33964798

[B39] Mackie G. (2018). Social norms change: believing makes it so. Soc. Res. Int. Q. 85, 141–166. doi: 10.1353/sor.2018.0008

[B40] Mackie G. Moneti F. Shakya H. Denny E. (2015). What Are Social Norms? How Are They Measured? UNICEF/UC San Diego Center on Global Justice. Available online at: https://justicegroup.org/wp-content/uploads/2021/12/4_09_30_Whole_What_are_Social_Norms.pdf (Accessed September 30, 2025).

[B41] Malhotra A. Elnakib S. (2021). 20 years of the evidence base on what works to prevent child marriage: a systematic review. J. Adol. Health 68, 847–862. doi: 10.1016/j.jadohealth.2020.11.017 33446401

[B42] Mazurana D. Marshak A. Spears K. (2019). Child marriage in armed conflict. Int. Rev. Red Cross 101, 575–601. doi: 10.1017/S1816383120000156

[B43] McDougal L. Jackson E. C. McClendon K. A. Belayneh Y. Sinha A. Raj A. (2018). Beyond the statistic: exploring the process of early marriage decision-making using qualitative findings from Ethiopia and India. BMC Women's Health 18:144. doi: 10.1186/s12905-018-0631-z 30143040 PMC6109273

[B44] Mieth K. Hasan T. Chakrabarty A. Lee K. Kaiser A. Hasan T. . (2025). “What other option did I have?” The effect of conflict and displacement on child marriage and early childbearing among displaced Rohingya adolescents. Confl. Health 19:16. doi: 10.1186/s13031-025-00656-2 40091116 PMC11912763

[B45] Ministry of Planning and International Cooperation (2020). Jordan Response Plan for the Syrian Crisis 2020–2022. Amman: Ministry of Planning and International Cooperation, Government of the Hashemite Kingdom of Jordan.

[B46] Mowri S. Sultana R. Biswas S. Azmi R. Ahsan S. Rashid F. (2020). “Binary framing of consent and coercion of child marriage: a critique,” in Dreaming of a Better Life: Child Marriage Through Adolescent Eyes, eds. Crivello G. Mann G. (Oxford: Young Lives), 21–32. Available online at: https://www.younglives.org.uk/sites/; www.younglives.org.uk/files/YL-DreamingOfBetterLife-LowRes_0.pdf (Accessed September 30, 2025).

[B47] Mukherjee A. Sekher T. V. (2017). “Do only girls suffer? We too!” Early marriage repercussions on boys in rural India. Econ. Polit. Wkly. 52, 75–82.

[B48] Murphy M. Arango D. Hill A. Contreras M. MacRae M. Ellsberg M. (2016). What Works to Prevent and Respond to Violence Against Women and Girls in Conflict and Humanitarian Settings? Evidence Brief. Washington, DC and London: George Washington University and International Rescue Committee.

[B49] Pearson I. Page S. Zimmerman C. Meinck F. Gennari F. Guedes A. . (2023). The co-occurrence of intimate partner violence and violence against children: a systematic review on associated factors in low- and middle-income countries. Trauma Violence Abuse 24, 2097–2114. doi: 10.1177/15248380221082943 35481390 PMC10486154

[B50] Pourtaheri A. Sany S. B. T. Aghaee M. A. Ahangari H. Peyman N. (2023). Prevalence and factors associated with child marriage, a systematic review. BMC Women's Health 23:531. doi: 10.1186/s12905-023-02634-3 37817117 PMC10565969

[B51] Psaki S. R. Melnikas A. J. Haque E. Saul G. Misunas C. Patel S. K. . (2021). What are the drivers of child marriage? A conceptual framework to guide policies and programs. J. Adol. Health 69, S13–S22. doi: 10.1016/j.jadohealth.2021.09.001 34809895

[B52] Rialet J. Greene M. E. Lauro G. (2022). Boyhood and Child, Early, and Forced Marriages and Unions: An Evidence Review. Washington, DC and New York, NY: Equimundo and United Nations Population Fund (UNFPA).

[B53] Shakya H. B. Silverman J. Barker K. M. Lapsansky C. Yore J. Aliou S. . (2020). Associations between village-level norms on marital age and marital choice outcomes among adolescent wives in rural Niger. SSM Popul. Health 11:100621. doi: 10.1016/j.ssmph.2020.100621 32685655 PMC7358742

[B54] Siddiqi M. Greene M. E. (2022). Mapping the field of child marriage: evidence, gaps, and future directions from a large-scale systematic scoping review, 2000–2019. J. Adolesc. Health 70, S9–S16. doi: 10.1016/j.jadohealth.2021.09.020 35184839

[B55] Singh R. Goli S. Singh A. (2022). Armed conflicts and girl child marriages: a global evidence. Child. Youth Serv. Rev. 137:106458. doi: 10.1016/j.childyouth.2022.106458

[B56] Sripad P. Pinchoff J. Dadi C. Dougherty L. (2024). Measuring social norms related to child marriage among married women and men in Niger. PLoS ONE 19:e0307595. doi: 10.1371/journal.pone.0307595 39058690 PMC11280256

[B57] Stark L. (2018). Early marriage and cultural constructions of adulthood in two slums in Dar es Salaam. Cult. Health Sex. 20, 888–901 doi: 10.1080/13691058.2017.1390162 29111880

[B58] Taylor A. Y. Murphy-Graham E. Van Horn J. Vaitla B. Del Valle Á. Cislaghi B. (2019). Child marriages and unions in Latin America: understanding the roles of agency and social norms. J. Adolesc. Health 64, S45–S51. doi: 10.1016/j.jadohealth.2018.12.017 30914168 PMC6426822

[B59] UNICEF (2023). Is an End to Child Marriage within Reach? Latest Trends and Future Prospects. New York, NY: UNICEF.

[B60] United Nations Children's Fund (UNICEF) (2014). A Study on Early Marriage in Jordan 2014. Amman: UNICEF Jordan.

[B61] United Nations General Assembly (2015). Transforming Our World: The 2030 Agenda for Sustainable Development. New York, NY: United Nations Department of Economic and Social Affairs.

[B62] United Nations High Commissioner for Refugees (UNHCR) (2022). Global Trends: Forced Displacement in 2022. Geneva: UNHCR.

[B63] Velloza J. Davies L. D. Ensminger A. L. Theofelus F. M. Andjamba H. Kamuingona R. . (2022). Cycles of violence among young women in Namibia: exploring the links between childhood violence and adult intimate partner violence from the Violence Against Children and Youth Survey. J. Interpersonal Violence 37, NP22992–NP23014. doi: 10.1177/08862605211073107 35156448 PMC9661872

[B64] Wahyuningsih S. Widati S. Puspitasari N. Salim L. A. Azkiya M. W. (2025). Exploring adolescent girls' involvement in decision-making processes regarding child marriage: a systematic review. Narra J. 5:1656. doi: 10.52225/narra.v5i1.1656 40352240 PMC12059853

[B65] Wodon Q. Male C. Nayihouba A. Onagoruwa A. Savadogo A. Yedan A. . (2017). Economic Impacts of Child Marriage: Global Synthesis Report (Conference Edition June 2017). Washington, DC: The World Bank and International Center for Research on Women (ICRW).

[B66] World Economic Forum (2024). Global Gender Gap Report 2024. Geneva: World Economic Forum. Available online at: https://www3.weforum.org/docs/WEF_GGGR_2024.pdf (Accessed October 5, 2025).

[B67] Zuntz A.-C. Palattiyil G. Amawi A. Al Akash R. Nashwan A. Al Majali A. . (2021). Early marriage and displacement – a conversation: how Syrian daughters, mothers and mothers-in-law in Jordan understand marital decision-making. J. British Acad. 9, 179–212. doi: 10.5871/jba/009.179

